# 1,3-Bis[4-(meth­oxy­carbon­yl)benz­yl]benzimidazolium bromide monohydrate

**DOI:** 10.1107/S1600536810041929

**Published:** 2010-11-06

**Authors:** Hua-Rong Huang, Guo Wen-Jiao, Zhi-Yun Du, Yan-Xiong Fang, Kun Zhang

**Affiliations:** a Guangdong University of Technology, Faculty of Chemical Engineering and Light Industry, Guangzhou 510006, Guangdong, People’s Republic of China

## Abstract

In the title compound, C_25_H_23_N_2_O_4_^+^·Br^−^·H_2_O, the dihedral angles between the benzimidazole ring system and the two benzene rings are 87.77 (11) and 63.05 (11)°; the dihedral angle between the two benzene rings is 66.25 (13)°. The crystal structure exhibits C—H⋯O and O—H⋯Br inter­actions; it is also stabilized by π–π stacking inter­actions, with a face-to-face separation of 3.456 Å between parallel benzimidazole ring systems.

## Related literature

For general background to and the therapeutic properties of benzimidazole derivatives, see: Herrmann (2002 ▶); Herrmann *et al.* (1995 ▶, 1998 ▶); Navarro *et al.* (2006 ▶). For related structures, see: Akkurt *et al.* (2005 ▶); Pınar *et al.* (2006 ▶); Arslan *et al.* (2009 ▶). For reference bond lengths, see: Allen *et al.* (1987 ▶).
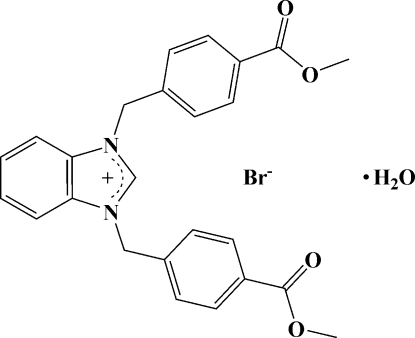


## Experimental

### 

#### Crystal data

C_25_H_23_N_2_O_4_^+^·Br^−^·H_2_O
*M* *_r_* = 513.38
Monoclinic, 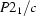

*a* = 13.604 (2) Å
*b* = 9.3537 (16) Å
*c* = 18.962 (3) Å
β = 107.006 (3)°
*V* = 2307.4 (7) Å^3^
*Z* = 4
Mo *K*α radiation
μ = 1.82 mm^−1^
*T* = 110 K
0.39 × 0.36 × 0.35 mm

#### Data collection

Bruker SMART CCD 1K area-detector diffractometer
Absorption correction: multi-scan (*SADABS*; Sheldrick, 1996 ▶) *T*_min_ = 0.537, *T*_max_ = 0.568
13524 measured reflections
5002 independent reflections
3422 reflections with *I* > 2σ(*I*)
*R*_int_ = 0.046

#### Refinement

*R*[*F* ^2^ > 2σ(*F* ^2^)] = 0.041
*wR*(*F* ^2^) = 0.096
*S* = 1.05
5002 reflections
300 parameters
H-atom parameters constrained
Δρ_max_ = 0.78 e Å^−3^
Δρ_min_ = −0.35 e Å^−3^

### 

Data collection: *SMART* (Bruker, 1999 ▶); cell refinement: *SAINT-Plus* (Bruker, 1999 ▶); data reduction: *SAINT-Plus*; program(s) used to solve structure: *SHELXS97* (Sheldrick, 2008 ▶); program(s) used to refine structure: *SHELXL97* (Sheldrick, 2008 ▶); molecular graphics: *SHELXTL* (Sheldrick, 2008 ▶); software used to prepare material for publication: *SHELXTL*.

## Supplementary Material

Crystal structure: contains datablocks global, I. DOI: 10.1107/S1600536810041929/wn2412sup1.cif

Structure factors: contains datablocks I. DOI: 10.1107/S1600536810041929/wn2412Isup2.hkl

Additional supplementary materials:  crystallographic information; 3D view; checkCIF report

## Figures and Tables

**Table 1:** Hydrogen-bond geometry (Å, °)

*D*—H⋯*A*	*D*—H	H⋯*A*	*D*⋯*A*	*D*—H⋯*A*
C1—H1⋯O1*W*	0.95	2.14	3.083 (3)	175
O1*W*—H1*A*⋯Br1	0.85	2.49	3.332 (2)	173
O1*W*—H1*B*⋯Br1^i^	0.84	2.43	3.269 (2)	173

Symmetry code: (i) 
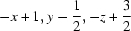
.

**Fig. 1.**
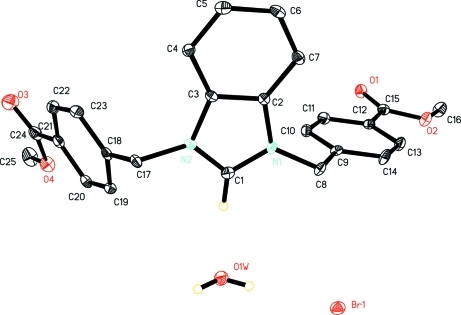
Perspective view showing 30% probability displacement ellipsoids and the atom-numbering scheme. Hydrogen atoms have been omitted.

**Fig. 2.**
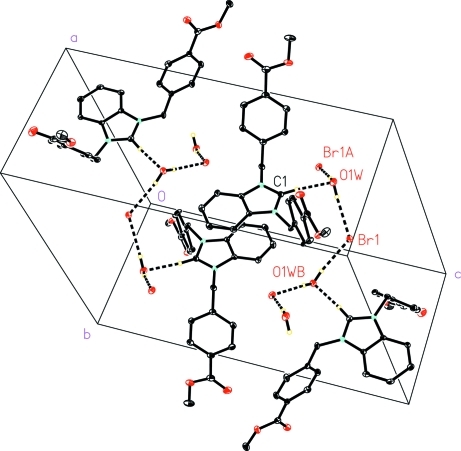
Crystal packing of the title compound. Dashed lines indicate hydrogen bonds. Symmetry: A = -x + 1, y - 1/2, -z + 3/2; B = -x + 1, y + 1/2, -z + 3/2. H atoms not involved in the hydrogen bond interactions have been omitted for clarity.

## supplementary crystallographic information

### Comment

As a new class of compounds in organometallic chemistry, N-heterocyclic carbenes have attracted much interest; they have often been applied as catalysts for Suzuki-Miyura, Sonogashira, Stille and Heck reactions (Herrmann, 2002; Herrmann *et al.*, 1995; Navarro *et al.*, 2006).

In this work, we report the structure of a new N-heterocyclic carbene derivative, 1,3-bis(4-(methoxycarbonyl)benzyl)benzimidazolium bromide monohydrate. The molecular structure of the title compound is depicted in Fig. 1. All bond lengths are in normal ranges (Allen *et al.*,1987).

The dihedral angles between the benzimidazole ring system and the two (C9–C14) and (C18–C23) benzene rings are 87.77 (11)° and 63.05 (11)°, respectively; the dihedral angle between the two benzene rings is 66.25 (13)°.

The bromide anions and water molecules form infinite *meso*-helical chains parallel to the *b* axis *via* O—H···Br hydrogen bonds, and the cations are linked to water molecules by C—H···O hydrogen bonds (Fig. 2, Table 1). In the crystal structure, π–π stacking interactions occurs between parallel benzimidazole rings, with a face-to-face separation of 3.456 Å (Fig.  2).

The crystal structures of three very similar benzimidazolium halide monohydrates have been published in recent years (Akkurt *et al.*, 2005; Pınar *et al.*, 2006; Arslan *et al.*, 2009;),

### Experimental

4-(Methoxycarbonyl)benzyl bromide (2.28 g, 10.0 mmol) was slowly added to a solution of benzimidazole (0.59 g, 5.0 mmol) in acetone (25 ml) and the resulting mixture was stirred under reflux for over a period of of 24 h. The volume of solvent was concentrated by *ca* 10 ml and then cooled to rt. The resulting precipitate was collected, washed with acetone, and dried to afford a white powder. The crude product was recrystallized from methanol/water. Yield 1.81 g, 73.2%. Elemental analysis, calcd (%) for C_25_H_25_N_2_BrO_5_: C 58.49, H 4.91; found(%): C 58.52, H 4.85. The FAB mass spectrum showed ions at 496.

### Refinement

The C-bound H atoms were positioned geometrically and were included in the refinement in the riding-model approximation, with distances 0.98 (CH_3_), 0.99 (CH_2_) and 0.95 Å (aromatic). The two water H atoms were located in a difference Fourier map and then refined as riding on the water O atom (0.84 and 0.85 Å). *U*_iso_(H) = x*U*_eq_(attached atom), where x = 1.5 for O and methyl C, 1.2 for all other C.

### Crystal data

**Table:**

C_25_H_23_N_2_O_4_^+^·Br^−^·H_2_O	*F*(000) = 1056
*M**_r_* = 513.38	*D*_x_ = 1.478 Mg m^−^^3^
Monoclinic, *P*2_1_/*c*	Mo *K*α radiation, λ = 0.71073 Å
Hall symbol: -P 2ybc	Cell parameters from 3740 reflections
*a* = 13.604 (2) Å	θ = 2.3–26.8°
*b* = 9.3537 (16) Å	µ = 1.82 mm^−^^1^
*c* = 18.962 (3) Å	*T* = 110 K
β = 107.006 (3)°	Block, colorless
*V* = 2307.4 (7) Å^3^	0.39 × 0.36 × 0.35 mm
*Z* = 4	

### Data collection

**Table:**

Bruker SMART CCD 1K area-detector diffractometer	5002 independent reflections
Radiation source: fine-focus sealed tube	3422 reflections with *I* > 2σ(*I*)
graphite	*R*_int_ = 0.046
φ and ω scans	θ_max_ = 27.1°, θ_min_ = 2.3°
Absorption correction: multi-scan (*SADABS*; Sheldrick, 1996)	*h* = −17→15
*T*_min_ = 0.537, *T*_max_ = 0.568	*k* = −11→11
13524 measured reflections	*l* = −18→24

### Refinement

**Table:**

Refinement on *F*^2^	Primary atom site location: structure-invariant direct methods
Least-squares matrix: full	Secondary atom site location: difference Fourier map
*R*[*F*^2^ > 2σ(*F*^2^)] = 0.041	Hydrogen site location: inferred from neighbouring sites
*wR*(*F*^2^) = 0.096	H-atom parameters constrained
*S* = 1.05	*w* = 1/[σ^2^(*F*_o_^2^) + (0.0417*P*)^2^ + 0.4447*P*] where *P* = (*F*_o_^2^ + 2*F*_c_^2^)/3
5002 reflections	(Δ/σ)_max_ < 0.001
300 parameters	Δρ_max_ = 0.78 e Å^−^^3^
0 restraints	Δρ_min_ = −0.35 e Å^−^^3^

### Special details

**Table:**

Geometry. All e.s.d.'s (except the e.s.d. in the dihedral angle between two l.s. planes) are estimated using the full covariance matrix. The cell e.s.d.'s are taken into account individually in the estimation of e.s.d.'s in distances, angles and torsion angles; correlations between e.s.d.'s in cell parameters are only used when they are defined by crystal symmetry. An approximate (isotropic) treatment of cell e.s.d.'s is used for estimating e.s.d.'s involving l.s. planes.
Refinement. Refinement of *F*^2^ against ALL reflections. The weighted *R*-factor *wR* and goodness of fit *S* are based on *F*^2^, conventional *R*-factors *R* are based on *F*, with *F* set to zero for negative *F*^2^. The threshold expression of *F*^2^ > σ(*F*^2^) is used only for calculating *R*-factors(gt) *etc*. and is not relevant to the choice of reflections for refinement. *R*-factors based on *F*^2^ are statistically about twice as large as those based on *F*, and *R*- factors based on ALL data will be even larger.

### Fractional atomic coordinates and isotropic or equivalent isotropic displacement parameters (Å^2^)

**Table:**

	*x*	*y*	*z*	*U*_iso_*/*U*_eq_	
Br1	0.51077 (2)	0.40648 (3)	0.834559 (16)	0.02439 (10)	
C1	0.6347 (2)	0.3275 (3)	0.63706 (15)	0.0175 (6)	
H1	0.6346	0.2667	0.6772	0.021*	
C2	0.6389 (2)	0.5212 (3)	0.57082 (15)	0.0145 (6)	
C3	0.63019 (19)	0.4021 (3)	0.52566 (14)	0.0137 (6)	
C4	0.6237 (2)	0.4155 (3)	0.45119 (15)	0.0178 (6)	
H4	0.6179	0.3347	0.4198	0.021*	
C5	0.6263 (2)	0.5528 (3)	0.42594 (16)	0.0233 (7)	
H5	0.6223	0.5668	0.3756	0.028*	
C6	0.6346 (2)	0.6733 (3)	0.47144 (16)	0.0214 (7)	
H6	0.6357	0.7658	0.4511	0.026*	
C7	0.6410 (2)	0.6601 (3)	0.54498 (16)	0.0201 (7)	
H7	0.6466	0.7410	0.5762	0.024*	
C8	0.6423 (2)	0.5565 (3)	0.70503 (15)	0.0200 (7)	
H8A	0.6190	0.4961	0.7399	0.024*	
H8B	0.5916	0.6346	0.6886	0.024*	
C9	0.7443 (2)	0.6214 (3)	0.74563 (15)	0.0175 (6)	
C10	0.8378 (2)	0.5707 (3)	0.74080 (15)	0.0198 (7)	
H10	0.8398	0.4923	0.7094	0.024*	
C11	0.9281 (2)	0.6342 (3)	0.78168 (16)	0.0209 (7)	
H11	0.9919	0.5990	0.7780	0.025*	
C12	0.9273 (2)	0.7487 (3)	0.82806 (15)	0.0172 (6)	
C13	0.8336 (2)	0.7982 (3)	0.83393 (16)	0.0229 (7)	
H13	0.8317	0.8746	0.8665	0.027*	
C14	0.7432 (2)	0.7356 (3)	0.79225 (16)	0.0237 (7)	
H14	0.6793	0.7713	0.7955	0.028*	
C15	1.0268 (2)	0.8163 (3)	0.86905 (16)	0.0202 (7)	
C16	1.1068 (2)	0.9863 (4)	0.96014 (18)	0.0311 (8)	
H16A	1.1500	0.9130	0.9913	0.047*	
H16B	1.0889	1.0592	0.9914	0.047*	
H16C	1.1443	1.0309	0.9290	0.047*	
C17	0.6111 (2)	0.1329 (3)	0.54368 (16)	0.0180 (6)	
H17A	0.5744	0.1315	0.4902	0.022*	
H17B	0.5669	0.0846	0.5696	0.022*	
C18	0.7096 (2)	0.0511 (3)	0.55708 (16)	0.0183 (6)	
C19	0.7559 (2)	−0.0094 (3)	0.62603 (16)	0.0198 (7)	
H19	0.7242	0.0011	0.6643	0.024*	
C20	0.8467 (2)	−0.0841 (3)	0.63950 (16)	0.0203 (6)	
H20	0.8774	−0.1250	0.6867	0.024*	
C21	0.8938 (2)	−0.0994 (3)	0.58324 (17)	0.0221 (7)	
C22	0.8476 (2)	−0.0409 (3)	0.51461 (17)	0.0246 (7)	
H22	0.8788	−0.0526	0.4762	0.029*	
C23	0.7567 (2)	0.0343 (3)	0.50129 (17)	0.0238 (7)	
H23	0.7260	0.0747	0.4540	0.029*	
C24	0.9937 (2)	−0.1754 (3)	0.5955 (2)	0.0281 (8)	
C25	1.1340 (2)	−0.2807 (4)	0.6830 (2)	0.0410 (9)	
H25A	1.1294	−0.3672	0.6531	0.061*	
H25B	1.1572	−0.3061	0.7354	0.061*	
H25C	1.1832	−0.2141	0.6720	0.061*	
N1	0.64248 (17)	0.4697 (2)	0.64073 (12)	0.0159 (5)	
N2	0.62711 (17)	0.2832 (2)	0.56895 (12)	0.0150 (5)	
O1	1.10918 (15)	0.7850 (2)	0.86183 (11)	0.0258 (5)	
O2	1.01338 (14)	0.9206 (2)	0.91378 (11)	0.0246 (5)	
O3	1.03392 (18)	−0.1982 (3)	0.54791 (14)	0.0406 (6)	
O4	1.03454 (16)	−0.2138 (2)	0.66612 (12)	0.0312 (5)	
O1W	0.63208 (16)	0.1475 (2)	0.77270 (11)	0.0273 (5)	
H1A	0.5960	0.2114	0.7851	0.041*	
H1B	0.5933	0.0829	0.7485	0.041*	

### Atomic displacement parameters (Å^2^)

**Table:**

	*U*^11^	*U*^22^	*U*^33^	*U*^12^	*U*^13^	*U*^23^
Br1	0.02588 (17)	0.02610 (17)	0.02259 (17)	0.00320 (15)	0.00927 (12)	0.00488 (14)
C1	0.0135 (15)	0.0212 (16)	0.0173 (16)	0.0022 (12)	0.0037 (12)	0.0028 (13)
C2	0.0114 (14)	0.0154 (15)	0.0157 (15)	−0.0003 (11)	0.0027 (11)	0.0001 (12)
C3	0.0089 (13)	0.0144 (13)	0.0162 (14)	−0.0020 (12)	0.0012 (10)	0.0015 (12)
C4	0.0184 (15)	0.0191 (15)	0.0160 (14)	−0.0049 (13)	0.0052 (11)	−0.0032 (13)
C5	0.0225 (16)	0.0270 (17)	0.0200 (16)	−0.0031 (13)	0.0054 (13)	0.0037 (13)
C6	0.0181 (16)	0.0179 (16)	0.0272 (17)	−0.0026 (13)	0.0054 (13)	0.0061 (13)
C7	0.0178 (16)	0.0163 (15)	0.0261 (17)	−0.0013 (12)	0.0065 (13)	−0.0001 (13)
C8	0.0220 (16)	0.0240 (16)	0.0159 (15)	0.0011 (13)	0.0087 (12)	−0.0054 (12)
C9	0.0186 (15)	0.0216 (16)	0.0140 (15)	−0.0008 (12)	0.0073 (12)	0.0011 (12)
C10	0.0236 (16)	0.0175 (15)	0.0183 (15)	0.0029 (13)	0.0062 (12)	−0.0007 (12)
C11	0.0163 (15)	0.0210 (16)	0.0259 (17)	0.0043 (12)	0.0069 (13)	0.0042 (13)
C12	0.0175 (15)	0.0193 (15)	0.0142 (14)	−0.0014 (12)	0.0040 (12)	0.0016 (12)
C13	0.0238 (17)	0.0246 (17)	0.0218 (17)	−0.0007 (13)	0.0091 (13)	−0.0083 (13)
C14	0.0150 (15)	0.0323 (18)	0.0256 (17)	0.0026 (13)	0.0084 (13)	−0.0106 (14)
C15	0.0177 (16)	0.0213 (16)	0.0213 (16)	0.0019 (13)	0.0051 (12)	0.0041 (13)
C16	0.0222 (18)	0.041 (2)	0.0300 (19)	−0.0085 (15)	0.0067 (14)	−0.0140 (16)
C17	0.0208 (16)	0.0119 (14)	0.0199 (16)	−0.0027 (12)	0.0039 (12)	−0.0001 (12)
C18	0.0184 (15)	0.0118 (14)	0.0246 (16)	−0.0042 (12)	0.0060 (12)	−0.0025 (12)
C19	0.0216 (16)	0.0175 (15)	0.0223 (16)	−0.0033 (13)	0.0097 (13)	−0.0007 (13)
C20	0.0196 (15)	0.0146 (14)	0.0248 (16)	−0.0036 (13)	0.0033 (12)	−0.0004 (13)
C21	0.0208 (15)	0.0135 (14)	0.0333 (18)	−0.0008 (13)	0.0100 (13)	−0.0006 (14)
C22	0.0268 (17)	0.0201 (16)	0.0306 (18)	−0.0003 (14)	0.0145 (14)	−0.0017 (14)
C23	0.0291 (18)	0.0193 (16)	0.0238 (17)	0.0008 (14)	0.0089 (14)	0.0044 (13)
C24	0.0225 (17)	0.0198 (17)	0.045 (2)	−0.0019 (14)	0.0142 (16)	0.0007 (15)
C25	0.0204 (18)	0.034 (2)	0.064 (3)	0.0094 (16)	0.0054 (17)	−0.0011 (19)
N1	0.0152 (13)	0.0175 (12)	0.0161 (13)	0.0005 (10)	0.0061 (10)	−0.0024 (10)
N2	0.0146 (12)	0.0133 (12)	0.0163 (13)	−0.0014 (10)	0.0029 (10)	0.0017 (10)
O1	0.0178 (12)	0.0253 (12)	0.0345 (13)	0.0012 (9)	0.0077 (9)	−0.0024 (10)
O2	0.0154 (10)	0.0332 (13)	0.0247 (11)	−0.0023 (10)	0.0050 (8)	−0.0103 (10)
O3	0.0364 (14)	0.0392 (15)	0.0547 (17)	0.0107 (12)	0.0267 (13)	0.0050 (12)
O4	0.0196 (11)	0.0291 (12)	0.0432 (15)	0.0067 (10)	0.0065 (10)	0.0029 (11)
O1W	0.0282 (12)	0.0296 (12)	0.0249 (12)	−0.0060 (10)	0.0092 (10)	−0.0058 (10)

### Geometric parameters (Å, °)

**Table:**

C1—N2	1.331 (3)	C15—O1	1.205 (3)
C1—N1	1.334 (3)	C15—O2	1.340 (3)
C1—H1	0.9500	C16—O2	1.453 (3)
C2—C3	1.389 (4)	C16—H16A	0.9800
C2—C7	1.392 (4)	C16—H16B	0.9800
C2—N1	1.398 (3)	C16—H16C	0.9800
C3—N2	1.390 (3)	C17—N2	1.481 (3)
C3—C4	1.394 (4)	C17—C18	1.499 (4)
C4—C5	1.375 (4)	C17—H17A	0.9900
C4—H4	0.9500	C17—H17B	0.9900
C5—C6	1.403 (4)	C18—C19	1.394 (4)
C5—H5	0.9500	C18—C23	1.396 (4)
C6—C7	1.377 (4)	C19—C20	1.378 (4)
C6—H6	0.9500	C19—H19	0.9500
C7—H7	0.9500	C20—C21	1.403 (4)
C8—N1	1.466 (3)	C20—H20	0.9500
C8—C9	1.504 (4)	C21—C22	1.382 (4)
C8—H8A	0.9900	C21—C24	1.490 (4)
C8—H8B	0.9900	C22—C23	1.381 (4)
C9—C10	1.385 (4)	C22—H22	0.9500
C9—C14	1.390 (4)	C23—H23	0.9500
C10—C11	1.381 (4)	C24—O3	1.204 (4)
C10—H10	0.9500	C24—O4	1.340 (4)
C11—C12	1.388 (4)	C25—O4	1.439 (4)
C11—H11	0.9500	C25—H25A	0.9800
C12—C13	1.391 (4)	C25—H25B	0.9800
C12—C15	1.490 (4)	C25—H25C	0.9800
C13—C14	1.382 (4)	O1W—H1A	0.8490
C13—H13	0.9500	O1W—H1B	0.8441
C14—H14	0.9500		
			
N2—C1—N1	110.0 (3)	O2—C16—H16A	109.5
N2—C1—H1	125.0	O2—C16—H16B	109.5
N1—C1—H1	125.0	H16A—C16—H16B	109.5
C3—C2—C7	122.6 (3)	O2—C16—H16C	109.5
C3—C2—N1	106.3 (2)	H16A—C16—H16C	109.5
C7—C2—N1	131.1 (3)	H16B—C16—H16C	109.5
C2—C3—N2	106.8 (2)	N2—C17—C18	112.9 (2)
C2—C3—C4	121.4 (2)	N2—C17—H17A	109.0
N2—C3—C4	131.8 (2)	C18—C17—H17A	109.0
C5—C4—C3	115.9 (3)	N2—C17—H17B	109.0
C5—C4—H4	122.1	C18—C17—H17B	109.0
C3—C4—H4	122.1	H17A—C17—H17B	107.8
C4—C5—C6	122.8 (3)	C19—C18—C23	118.9 (3)
C4—C5—H5	118.6	C19—C18—C17	120.1 (3)
C6—C5—H5	118.6	C23—C18—C17	121.0 (3)
C7—C6—C5	121.4 (3)	C20—C19—C18	120.9 (3)
C7—C6—H6	119.3	C20—C19—H19	119.5
C5—C6—H6	119.3	C18—C19—H19	119.5
C6—C7—C2	116.0 (3)	C19—C20—C21	119.7 (3)
C6—C7—H7	122.0	C19—C20—H20	120.2
C2—C7—H7	122.0	C21—C20—H20	120.2
N1—C8—C9	115.1 (2)	C22—C21—C20	119.5 (3)
N1—C8—H8A	108.5	C22—C21—C24	118.6 (3)
C9—C8—H8A	108.5	C20—C21—C24	121.9 (3)
N1—C8—H8B	108.5	C23—C22—C21	120.7 (3)
C9—C8—H8B	108.5	C23—C22—H22	119.7
H8A—C8—H8B	107.5	C21—C22—H22	119.7
C10—C9—C14	119.1 (3)	C22—C23—C18	120.2 (3)
C10—C9—C8	123.8 (3)	C22—C23—H23	119.9
C14—C9—C8	117.1 (2)	C18—C23—H23	119.9
C11—C10—C9	120.0 (3)	O3—C24—O4	123.7 (3)
C11—C10—H10	120.0	O3—C24—C21	124.2 (3)
C9—C10—H10	120.0	O4—C24—C21	112.2 (3)
C10—C11—C12	121.1 (3)	O4—C25—H25A	109.5
C10—C11—H11	119.4	O4—C25—H25B	109.5
C12—C11—H11	119.4	H25A—C25—H25B	109.5
C11—C12—C13	119.0 (3)	O4—C25—H25C	109.5
C11—C12—C15	118.9 (3)	H25A—C25—H25C	109.5
C13—C12—C15	122.1 (3)	H25B—C25—H25C	109.5
C14—C13—C12	119.8 (3)	C1—N1—C2	108.3 (2)
C14—C13—H13	120.1	C1—N1—C8	125.2 (2)
C12—C13—H13	120.1	C2—N1—C8	126.1 (2)
C13—C14—C9	121.0 (3)	C1—N2—C3	108.5 (2)
C13—C14—H14	119.5	C1—N2—C17	124.8 (2)
C9—C14—H14	119.5	C3—N2—C17	126.5 (2)
O1—C15—O2	123.5 (3)	C15—O2—C16	115.7 (2)
O1—C15—C12	124.8 (3)	C24—O4—C25	115.3 (3)
O2—C15—C12	111.6 (2)	H1A—O1W—H1B	109.6
			
C7—C2—C3—N2	−178.0 (2)	C19—C20—C21—C22	−0.9 (4)
N1—C2—C3—N2	0.9 (3)	C19—C20—C21—C24	178.2 (3)
C7—C2—C3—C4	0.5 (4)	C20—C21—C22—C23	1.1 (4)
N1—C2—C3—C4	179.4 (2)	C24—C21—C22—C23	−178.0 (3)
C2—C3—C4—C5	−0.1 (4)	C21—C22—C23—C18	−0.6 (4)
N2—C3—C4—C5	177.9 (3)	C19—C18—C23—C22	−0.2 (4)
C3—C4—C5—C6	−0.2 (4)	C17—C18—C23—C22	179.8 (3)
C4—C5—C6—C7	0.2 (4)	C22—C21—C24—O3	−5.4 (5)
C5—C6—C7—C2	0.1 (4)	C20—C21—C24—O3	175.5 (3)
C3—C2—C7—C6	−0.4 (4)	C22—C21—C24—O4	173.7 (3)
N1—C2—C7—C6	−179.1 (3)	C20—C21—C24—O4	−5.3 (4)
N1—C8—C9—C10	−20.6 (4)	N2—C1—N1—C2	0.5 (3)
N1—C8—C9—C14	161.6 (3)	N2—C1—N1—C8	173.9 (2)
C14—C9—C10—C11	−0.4 (4)	C3—C2—N1—C1	−0.9 (3)
C8—C9—C10—C11	−178.1 (3)	C7—C2—N1—C1	177.9 (3)
C9—C10—C11—C12	0.1 (4)	C3—C2—N1—C8	−174.2 (2)
C10—C11—C12—C13	1.0 (4)	C7—C2—N1—C8	4.6 (5)
C10—C11—C12—C15	−178.0 (3)	C9—C8—N1—C1	108.2 (3)
C11—C12—C13—C14	−1.8 (4)	C9—C8—N1—C2	−79.5 (3)
C15—C12—C13—C14	177.2 (3)	N1—C1—N2—C3	0.1 (3)
C12—C13—C14—C9	1.6 (5)	N1—C1—N2—C17	−176.2 (2)
C10—C9—C14—C13	−0.5 (4)	C2—C3—N2—C1	−0.7 (3)
C8—C9—C14—C13	177.4 (3)	C4—C3—N2—C1	−178.9 (3)
C11—C12—C15—O1	4.8 (4)	C2—C3—N2—C17	175.5 (2)
C13—C12—C15—O1	−174.2 (3)	C4—C3—N2—C17	−2.7 (5)
C11—C12—C15—O2	−177.2 (2)	C18—C17—N2—C1	−86.9 (3)
C13—C12—C15—O2	3.9 (4)	C18—C17—N2—C3	97.5 (3)
N2—C17—C18—C19	83.0 (3)	O1—C15—O2—C16	−6.2 (4)
N2—C17—C18—C23	−96.9 (3)	C12—C15—O2—C16	175.7 (2)
C23—C18—C19—C20	0.4 (4)	O3—C24—O4—C25	2.5 (5)
C17—C18—C19—C20	−179.6 (3)	C21—C24—O4—C25	−176.6 (3)
C18—C19—C20—C21	0.2 (4)		

### Hydrogen-bond geometry (Å, °)

**Table:**

*D*—H···*A*	*D*—H	H···*A*	*D*···*A*	*D*—H···*A*
C1—H1···O1W	0.95	2.14	3.083 (3)	175.
O1W—H1A···Br1	0.85	2.49	3.332 (2)	173.
O1W—H1B···Br1^i^	0.84	2.43	3.269 (2)	173.

Symmetry codes: (i) −*x*+1, *y*−1/2, −*z*+3/2.

## References

[bb1] Akkurt, M., Karaca, S., Küçükbay, H., Orhan, E. & Büyükgüngör, O. (2005). *Acta Cryst.* E**61**, o2452–o2454.

[bb2] Allen, F. H., Kennard, O., Watson, D. G., Brammer, L., Orpen, A. G. & Taylor, R. (1987). *J. Chem. Soc. Perkin Trans. 2*, pp. S1–19.

[bb3] Arslan, H., VanDerveer, D., Demir, S., Özdemir, İ. & Çetinkaya, B. (2009). *Acta Cryst.* E**65**, o208–o209. 10.1107/S1600536808043250 PMC2968113 21581662

[bb4] Bruker (1999). *SMART* and *SAINT-Plus* Bruker AXS Inc, Madison, Wisconsin, USA.

[bb5] Herrmann, W. A. (2002). *Angew. Chem. Int. Ed.* **41**, 1290–1309.

[bb6] Herrmann, W. A., Elison, M., Fischer, J., Köcher, C. & Artus, G. R. J. (1995). *Angew. Chem. Int. Ed. Engl.* **34**, 2371–2374.

[bb7] Herrmann, W. A., Reisinger, C. P. & Spiegler, M. (1998). *J. Organomet. Chem.* **557**, 93–96.

[bb8] Navarro, O., Marion, N., Oonishi, Y., Kelly, R. A. & Nolan, S. P. (2006). *J. Org. Chem.* **71**, 685–692. 10.1021/jo0521201 16408981

[bb9] Pınar, Ş., Akkurt, M., Küçükbay, H., Şireci, N. & Büyükgüngör, O. (2006). *Acta Cryst.* E**62**, o2223–o2225.

[bb10] Sheldrick, G. M. (1996). *SADABS* University of Göttingen, Germany.

[bb11] Sheldrick, G. M. (2008). *Acta Cryst.* A**64**, 112–122. 10.1107/S0108767307043930 18156677

